# Cruciferous Vegetables and Their Bioactive Metabolites: from Prevention to Novel Therapies of Colorectal Cancer

**DOI:** 10.1155/2022/1534083

**Published:** 2022-04-11

**Authors:** Duygu Ağagündüz, Teslime Özge Şahin, Birsen Yılmaz, Kübra Damla Ekenci, Şehriban Duyar Özer, Raffaele Capasso

**Affiliations:** ^1^Gazi University, Department of Nutrition and Dietetics, Ankara 06490, Turkey; ^2^Çukurova University, Department of Nutrition and Dietetics, Adana 01330, Turkey; ^3^Bandırma Onyedi Eylül University, Department of Nutrition and Dietetics, Bandırma, Balıkesir 10200, Turkey; ^4^University of Naples Federico II, Department of Agricultural Sciences, 80055 Naples, Italy

## Abstract

The *Brassicaceae* family, known as cruciferous vegetables, includes many economically important species, mainly edible oil plants, vegetable species, spice plants, and feed plants. Cruciferous vegetables are foods rich in nutritive composition and are also a good source of dietary fiber. Besides, cruciferous vegetables contain various bioactive chemicals known as glucosinolates and S-methyl cysteine sulfoxide, including sulphur-containing cancer-protective chemicals. Numerous studies have reported that daily intake of sulphurous vegetables helps prevent cancer formation and reduces cancer incidence, especially in colorectal cancer, through various mechanisms. The potential mechanisms of these compounds in preventing cancer in experimental studies are as follows: protecting cells against DNA damage, inactivating carcinogenic substances, showing antiviral and antibacterial effects, triggering apoptosis in cells with disrupted structure, inhibiting tumour cell migration causing metastasis and the development of tumour-feeding vessels (angiogenesis). These beneficial anticancer effects of cruciferous vegetables are generally associated with glucosinolates in their composition and some secondary metabolites, as well as other phenolic compounds, seed oils, and dietary fiber in the literature. This review aims to examine to the roles of cruciferous vegetables and their important bioactive metabolites in the prevention and treatment of colorectal cancer.

## 1. Introduction

Cancer is one of the leading causes of mortality in humans. According to the global burden of disease systematic analysis report, cancer caused the deaths of approximately 8.7 million people [1]. The death rate from cancer and cancer-related diseases is expected to double by 2030. Negative changes in people's lifestyles and environmental pollution stand out as the main causes of cancer [2]. Approximately one-third of the risk of colorectal cancer has been associated with red meat, processed food, and similar dietary habits [3]. Fruit and vegetable consumption is essential to prevent factors that cause colon cancer [4]. The World Cancer Research Fund (WCRF)/American Institute for Cancer Research (AICR) Third Expert Report stated that there is strong evidence that consuming plant foods containing dietary fibre such as plant foods decreases the risk of colorectal cancer [5]. In addition, it is a well-known fact that there is a negative correlation between vegetable consumption and the risk of colorectal cancer [6, 7]. Especially the bioactive components contained in plants have a potential role in preventing cancer and reducing the risk of cancer [8].

Many epidemiological studies have reported that daily intake of sulphurous vegetables helps prevent cancer formation and reduces cancer incidence [9, 10]. The *Brassicaceae* family, known as cruciferous vegetables, includes many economically important species, mainly edible oil plants, vegetable species, spice plants, and feed plants [11]. Vegetables such as broccoli, Brussels sprouts, cabbage, cauliflower, kale, radish, and turnip belong to the cruciferous family [12]. Vegetables in the cruciferous family, in other words, the edible roots of various plants from the mustard family, which belong to the *Brassica* genus, are also called *Brassica* (field mustard) vegetables. Vegetables including arugula, Chinese cabbage, horseradish, kohlrabi radish, watercress, and wasabi are also included in this family. They are consumed in the diet in the form of a fresh salad, steamed, or vegetable meal. [12, 13]. Vegetables in the cruciferous family contain chemicals known as glucosinolates and S-methyl cysteine sulfoxide, including sulphur-containing cancer-protective chemicals. Glucosinolates, mainly in the *Brassicaceae* family, are secondary metabolites that are economically valuable and have beneficial effects on human health. During the preparation, chewing, and digestion of foods, sulphurous vegetables are broken down to form biologically active compounds such as glucosinolates, indoles, nitriles, thiocyanates, and isothiocyanates [14]. Indole-3-carbinol (an indole) and sulforaphane (a type of isothiocyanate) are the compounds most frequently searched for anticancer effects. Cruciferous vegetables are also rich in various carotenoids (beta-carotene, lutein, and zeaxanthin), flavonoids, anthocyanins, coumarins, therapeutic antioxidant enzymes, terpenes, vitamins C, E, K, and folate, and minerals such as potassium, calcium, and selenium, and are a good source of fiber [14]. Research on various organs of rats and mice, including the bladder, breast, colon, liver, lung, and stomach, has reported that indoles and isothiocyanates inhibit the growth of cancer in these organs [15, 16]. The potential effects of these compounds in preventing cancer in studies conducted in laboratory animals and cell culture studies under laboratory conditions are as follows: protecting cells against DNA damage, inactivating carcinogenic substances, and triggering apoptosis (cell death) in cells with disrupted structure. These compounds also have therapeutic effects such as inhibiting tumour cell migration causing metastasis and the development of tumour-feeding vessels (angiogenesis). However, the results of research on humans are contradictory [4, 9, 10, 17]. Several case-control studies have shown that specific forms of the gene encoding glutathione S-transferase, the enzyme that metabolizes isothiocyanates and aids their elimination from the body, may influence the association between cruciferous vegetable intake and the risk of colorectal cancer in humans [18, 19]. In addition to their anticancer activities, it is reported that there are promising developments regarding the use of these vegetables and their bioactive metabolites in cancer treatment in line with the latest technological developments [20, 21].

This review aims to introduce and comprehensively examine to the roles of cruciferous, in other words, sulphurous vegetables and their bioactive metabolites in the prevention and treatment of colorectal cancer.

## 2. Chemical Composition of Cruciferous Vegetables

Cruciferous vegetables contain carbohydrates, protein, vitamin C, folic acid, vitamin A, iron, calcium, copper, selenium, and zinc. In addition to nutritive ingredients, these vegetables are also abundant in secondary metabolites, which have been shown to have positive effects on health, especially cancer [22, 23].

### 2.1. Energy and Nutritive Composition

Cruciferous vegetables are foods rich in nutritive composition and are also a good source of fiber. The botanical classifications of different cruciferous vegetables along with their dietary nomenclature are shown in Table 1 [24].

Energy and nutritional values of cruciferous vegetables are shown in Tables 2 [25] and 3 [25–31]. The energy density of these nutrients (85–95%), of which water constitutes a large part of their composition, is low (ranging between 11–48 kcal/100 g). Among the cruciferous vegetables, the content of kale is richer in fiber (4.1 g/100 g), calcium (254 mg/100 g), and iron (1.6 mg/100 g) than other vegetables. In addition to kale, collards (232 mg/100 g), arugula (160 mg/100 g), and Chinese cabbage (105 mg/100 g) contain high levels of calcium [25]. Cruciferous vegetables are good dietary sources of phosphorus, magnesium, potassium as well as calcium, and iron [32]. Vitamins are another major component of cruciferous vegetables, which are particularly rich in vitamin C, folic acid, carotenes, and vitamin K. Kale (93.4 mg/100 g) and broccoli (91.3 mg/100 g) have higher vitamin C content, while collards have higher folate content (129 *μ*g/100 g) than other vegetables. Moreover, collards and kale also contain high levels of beta-carotene and lutein + zeaxanthin [25, 33].

### 2.2. Secondary Metabolites

The term “secondary” was first used by A. Kossel in 1891, and it was emphasized that although primary metabolites are present in every living cell that can divide, secondary metabolites are present by chance and are not crucial to the life of an organism [34].

The absence of secondary compounds does not shorten the life of the organism, and they are not considered essential nutrients like other macro and micronutrients [35]. It is reported that there are over two million secondary metabolites, and these metabolites are generally classified according to their wide variety in structure, function, and biosynthesis [34]. Although phytochemicals are responsible for giving color, taste, smell, and taste to plants, the bioactive compounds found in cruciferous vegetables have also been shown to have many positive effects on human health [36, 37].

The main bioactive components found in cruciferous vegetables are glucosinolates (the breakdown products of glucosinolates such as isothiocyanates and indoles); phenolic compounds including flavonoids, hydroxycinnamic acids, anthocyanins and lignans; tocopherols (*α*-*δ*- and *γ*-tocopherols) and carotenoids (*α*-carotenoids with lutein, *β*-carotene); and fatty acids (palmitic, stearic, oleic, linoleic, linolenic, eicosenoic, and erucic acids). The amount of these bioactive ingredients varies according to the type of cruciferous vegetable [33]. In cruciferous vegetables, nitrogen-containing compounds constitute the major class of secondary metabolites, while glucosinolates and S-methyl cysteine sulfoxide are the major sulfur compounds. In total, 130 types of glucosinolates from 11 plant families have been identified, but only five of them (glucobrassicin, sinigrin, glucoraphasatin, glucoraphanin, and glucoiberin) are commonly found in foods [38, 39]. Glucosinolates are found in almost every member of the *Cruciferae* family. These sulfur-containing compounds especially isothiocyanates are responsible for the pungent aroma and bitter taste that distinguish cruciferous vegetables from other vegetables. The primary food sources of glucosinolates in humans are cruciferous vegetables including broccoli, cauliflower, cabbage, Chinese cabbage, Brussels sprouts, collard, kale, kohlrabi, and turnips [24]. Although the glucosinolate concentration of these vegetables is variable, it constitutes approximately 1% of their dry weight [40]. Different broccoli extracts and strains can have very different glucosinolate profiles. A glucosinolate, which is high in some species, may not be found in other species. Barbieri et al. reported that the highest levels of glucosinolates in different broccoli samples were glucobrassicin, neoglucobrassicin, and glucoraphanin, respectively [41]. On the other hand, the glucosinolate profile may vary depending on the type of plant [27]. For example, it is stated that broccoli is a good source of glucoraphanin and glucobrassicin, while watercress is a rich source of gluconasturin [42].

Glucosinolates are hydrolysed by the myrosinase enzyme found in plant tissues and converted into biologically active compounds such as indoles (indole-3-carbinol) and isothiocyanates (sulforaphane), which have been shown to have anticarcinogenic properties in vitro and animal studies [43]. Phenolic compounds are a group of phytochemicals produced as secondary metabolites by the shikimic acid pathway in plants and include a wide range of compounds [44]. These compounds can be classified according to the number and arrangement of carbon atoms in flavonoids (flavonols, flavones, flavan-3-ols, anthocyanidins, flavanones, isoflavones, and others) and nonflavonoids (phenolic acids, hydroxycinnamates, stilbenes, and others) [45]. The most common phenolic compounds found in cruciferous vegetables are tannins, phenolic acids, anthocyanidins, flavonols, coumarins, and flavones, and these are the main components that affect the antioxidant capacity of these vegetables [26]. Li et al. analyzed a total of 74 phenolic substances (including 16 hydroxycinnamic acids and derivatives; 58 flavonoids and derivatives) in 12 cruciferous vegetables, and the major flavonoids found in these vegetables were glycosylated quercetin, kaempferol, and isorhamnetin, and the main hydroxycinnamic acids were ferulic, sinapic, caffeic, and p-coumaric acids [46]. While the total phenolic content of watercress and cauliflower was higher than other vegetables, the concentration of transresveratrol was found at the highest level in arugula leaves (84 *μ*g/g dry weight). The strongest antioxidant compounds were determined as (−)-epicatechin, allyl isothiocyanate, kaempferol, ferulic acid, pterostilbene, quercetin-3-glucoside, quercetin-3-galactoside, and myricetin, respectively [26]. Phytosterols, tocopherols, and other terpenoids are other bioactive compounds in cruciferous vegetables, and their amounts may vary by type of vegetable [47]. The literature cites that plant sterols (phytosterols) have a wide variety of biological activities and beneficial effects on human health [48]. It is reported that while there is no significant difference in sterol content between raw and cooked vegetables, storing vegetables in a controlled atmosphere prevents sterol degradation compared to storage under normal conditions [47, 49]. The use of a controlled atmosphere is recommended to benefit from phytochemicals, especially sterols found in cruciferous vegetables [49].

### 2.3. Cruciferous Vegetables and Colorectal Cancer

Epidemiological evidence obtained from both prospective cohort studies and retrospective case-control studies conducted in recent years has shown an inverse relationship between the consumption of cruciferous vegetables and the risk of lung, stomach, colorectal, breast, bladder, and prostate cancer as well as myocardial infarction. These beneficial effects of cruciferous vegetables are generally associated with glucosinolates in their composition and degradation products [50].

Table 4 summarizes some epidemiological studies on cruciferous vegetables. In both long and short-term studies, cruciferous vegetables have been shown to reduce the risk of colorectal cancer in general. While consumption of these vegetables at least once a week is particularly associated with reduced colorectal cancer risk [51] specifically broccoli, cauliflower, and cabbage have been reported to be more effective vegetables in reducing cancer risk [52]. Although different results were obtained in analyses by gender, consumption of cruciferous vegetables in women is more closely associated with a reduced risk of colon cancer [17, 53]. However, with regard to colorectal cancer, cruciferous vegetables can both increase [17] and decrease the risk [54]. According to Singapore Chinese Health Study results, the colorectal cancer preventive activity of cruciferous vegetables is strongest among individuals deficient for GST. Because this isa major metabolic pathway for the elimination of major chemopreventive compounds [23], in summary, various factors such as gender, type, and composition of vegetables, and genetic characteristics of individuals can affect the colorectal cancer preventive properties of cruciferous vegetables. In this review, the bioactive components of cruciferous vegetables which were the most discussed in the literature regarding colorectal cancer and which were also important in terms of nutrition were examined and divided into special subheadings.

## 3. Glucosinolates

### 3.1. Chemical Structure

Glucosinolates (*β*-thioglucoside N-hydroxysulfates) are sulphur-rich, anionic, and water-soluble secondary metabolites found in cruciferous vegetables [59]. In its basic structure (Table 5), there is an amino acid-containing side chain that is in a sulphated isothiocyanate (ITC) group linked to D-thioglucose [59]. Glucosinolates are divided into 3 groups according to the different amino acid precursors in their side chains: aliphatic (methionine, isoleucine, leucine, or valine), aromatic (phenylalanine and tyrosine), and indole (tryptophan) [27]. Various processes such as the elongation of amino acids and side chain modifications lead to the synthesis of more than 200 glucosinolate species in cruciferous vegetables. However, only a few bioactive products of glucosinolates have been reported to have beneficial effects on human and animal health [60].

The main role of glucosinolates in plant physiology is to defend the plant. As a result of damage to plant tissues, glucosinolates undergo enzymatic hydrolysis and turn into bioactive compounds that are toxic to herbivores and pathogens [62]. Myrosinase (*β*-thioglucosidase) enzyme, which catalyses enzymatic hydrolysis, is found in separate compartments from glucosinolates in intact plant tissues to prevent plants from being affected by their toxicity [62, 63]. Due to the disruption of tissue integrity, the myrosinase enzyme encounters glucosinolates and the thioglucosidic bond in the structure of glucosinolates is hydrolyzed. As a result, glucose and an unstable aglycone, thiohydroxamate-O-sulfonate, are formed [63]. Thiohydroxamate-O-sulfonate undergoes spontaneous rearrangement depending on ambient conditions and turns into ITCs, thiocyanates, nitriles, epithionitriles, oxazolidine-2 thions, or indolyl compounds, which are bioactive products of glucosinolates [60, 63]. In addition to the contribution of these compounds to the health of the plant, studies have shown that they have various health benefits in humans, including anticancer, antibacterial, antidiabetic, antiobesity, antioxidant, and antimutagenic [37, 62].

### 3.2. Factors Affecting the Glucosinolate Content of Cruciferous Vegetables

All the processes that the cruciferous vegetables go through from the field to the final consumer have a vital effect on human health, and these processes influence the glucosinolate concentration and bioavailability [24]. The glucosinolate content of cruciferous vegetables can differ significantly between plants belonging to the same species, even between different parts of the same plant and the growth stages of the plants. It is generally stated that seeds contain higher levels of glucosinolate than other parts of the plant and younger shoots than mature plants. The glucosinolate content in a particular type of vegetable is greatly influenced by growing conditions (soil and climate), postharvest processing, and food processing [27]. Mithen et al. noted in a study in which they examined broccoli samples that the glucosinolate content of vegetables grown in soils with high sulphur and nitrogen content was significantly higher [64]. When the effect of the growth temperature of the plant is examined, it is seen that the glucosinolate levels are lower in plants grown at low temperatures. Therefore, plants harvested in winter or autumn have lower GLS levels than those harvested in spring or summer [65]. While all glucosinolates are similarly affected by postharvest processing conditions, environmental conditions such as stressful growing conditions commonly cause an increase in the level of indolic glucosinolates [27]. Food preparation, cooking, and storage methods are other critical factors affecting the glucosinolate content of vegetables. The method of preparation can make a huge difference both in the concentrations of glucosinolates from vegetables and in the bioavailability of their degradation products [66]. Most of the glucosinolates are resistant to chemical and thermal changes and hydrolysis, but only enzymatically [63]. However, the thermal degradation that occurs in the plant tissue with the cooking process can cause glucosinolates to pass into the water and cause a decrease in the glucosinolate concentration in the plant tissue, and it can prevent the conversion of glucosinolate to compounds such as ITC and indole-3-carbinol, which are biologically active metabolites by inhibiting myrosinase enzyme activity (at temperatures of 60°C and above) [66]. Therefore, thermal effects may affect, although not directly, the hydrolysis, bioavailability, and concentration of glucosinolates in vegetables. Various studies have shown that cooking in boiling water has a high level of glucosinolate losses (57–81%), supporting this information and that the losses are lower in steam cooking [67]. It has been found that there are high losses (64%) in boiling water and high-pressure cooking methods, where maximum conservation of glucosinolates is provided by steam cooking, microwave, and stir-frying methods [27, 68]. In addition to the cooking method, the cooking time also affects the glucosinolate concentration and bioavailability. One study showed that boiling broccoli, cabbage, cauliflower, and Brussels sprouts for 30 minutes led to a progressive decrease in glucosinolate concentration. However, there was no significant difference in glucosinolate concentrations from baseline in 0–20 minutes of steaming, 0–3 minutes of microwave cooking, and 0–5 minutes of stir-frying [69]. The losses caused by cooking also differ according to the type of vegetable. For example, while the glucosinolate loss occurring as a result of boiling broccoli and cauliflower for 30 minutes is 75–77%, it is 58% in Brussels sprouts [69]. Investigation of the effect of storage conditions on the glucosinolate composition of vegetables showed that the glucosinolate concentration of vegetables stored at room temperature for 5 days decreased by about 80% compared to fresh vegetables, and this rate was between 11 and 27% after 5–7 days of storage in a domestic refrigerator. Therefore, it is stated that the most appropriate storage temperature in terms of glucosinolate concentrations is the refrigerator temperature [65, 69, 70].

### 3.3. Metabolism and Bioavailability

After consumption of glucosinolate-containing foods, most of these compounds are metabolized in the intestinal lumen, while a small part is absorbed unchanged from the gastrointestinal mucosa. When these foods are consumed raw without processing, glucosinolates are hydrolyzed by the myrosinase enzyme in the food's own structure in the proximal part of the gastrointestinal tract to form thiohydroxamate-O-sulfonate, and this aglycone structure is rearranged to form sulfate ions and various bioactive metabolites such as ITCs, thiocyanates, nitriles, epithionitriles, and oxazolidin-2-thions [70]. In the formation of these bioactive metabolites, the presence of acidic pH in the environment, the presence of ferrous iron ions (Fe2+), and a special plant protein, epithiospesifier protein, lead to the formation of nitriles or epithionitriles [70]. In a neutral pH environment, the formation of ITCs is observed. If the side chain of the formed ITCs has a *β*-hydroxyl function, the ITCs will spontaneously cyclize to oxazolidine-2-thione. Other ITCs are unstable and readily dissociate into thiocyanate ions and indole-3-carbinol [65].

When these foods are cooked before consumption, myrosinase is denatured, and glucosinolates pass into the colon to be hydrolysed by the gut microbiota [70]. It is emphasized that the bacterial microflora of the human colon shows some myrosinase activity. In a study, the conversion rate of glucosinolates to ITCs was compared as a result of the consumption of 350 g (475 *μ*mol glucosinolate) watercress inactivated by heat treatment or 150 g (972 *μ*mol glucosinolate) raw watercress that preserved myrosinase activity. While the rate of ITC return was found to be 17.2–77.7% in those who consumed it raw, it varied between 1.2–7.3% in those who consumed it cooked [71]. In conclusion, the bioavailability of glucosinolates is adversely affected by the consumption of foods that do not contain myrosinase. Absorbed ITCs are conjugated to glutathione in the liver, and 12–80% of the amount taken from food is excreted in the urine as mercapturic acid (N-acetyl-S-(N-alkylthiocarbamoyl)-1-cysteine). Therefore, urinary excretion of mercapturic acid is used as a biomarker of ingested and metabolized ITCs [70]. The mercapturic acid level in the urine can be detected 2-3 hours after the ingestion of glucosinolate with food [66]. Like ITCs, nitriles, and epithionitriles can be excreted in the urine as mercapturic acids, while oxazolidin-2-thione and thiocyanate ion are excreted directly [70].

### 3.4. Health Promotion, Cancer Prevention, and Potential Therapeutic Roles

Cancer is defined as a genetic disease caused by changes in genes that control the functions of cells like growth and division. In addition to being inherited from parents, genetic changes that cause cancer can also occur because of errors that occur when cells divide or due to DNA damage caused by some environmental exposures [72].

It is highlighted that colorectal cancer is the third most common cancer type in men and the second in women worldwide and constitutes 9% of all cancer incidence [73]. The incidence of colorectal cancer varies greatly between geographical regions, with approximately 55% of cases occurring in developed countries. These geographical differences are due to lifestyle factors (such as physical inactivity, unhealthy diet, smoking, and obesity) accompanied by genetic predisposition and environmental exposures [73].

Cruciferous vegetables contain many bioactive components such as folate, vitamin C, tocopherols, carotenoids, and polyphenols. However, the anticarcinogenic effects of these vegetables are mostly associated with their glucosinolates [74]. The literature reports that ITCs, which are bioactive metabolites of glucosinolates, have high anticancer properties. Therefore, the anticarcinogenic properties of cruciferous vegetables are affected by the concentrations of glucosinolates in their structure, the conversion of these glucosinolates to ITCs, and the bioavailability of the ITC metabolite [69]. It is indicated that ITCs show their anticarcinogenic effects by blocking the cell cycle, stimulating apoptosis and autophagy, showing anti-inflammatory effects as a result of inhibition of the NF-*κ*B pathway, and activating detoxifying enzymes [60, 75]. The effects of glucosinolates on colorectal cancer have been studied in many experimental animal models and *cell culture models*. These studies are summarized in Tables 6 and 7.

### 3.5. Activation of Detoxification Enzymes

Carcinogens taken in the diet and exposed to environmental factors are metabolized by phase 1 and phase 2 biotransformation enzymes, especially after they enter the circulation. In phase 1 metabolism, procarcinogens undergo oxidation via cytochrome p450 (CYP) enzymes and turn into more polar and active compounds [50, 65]. The products of phase 1 metabolism are highly reactive intermediates that can be harmful by binding to critical macromolecules such as DNA, RNA, and protein. On the other hand, during phase 2 metabolism, carcinogens are converted to inactive metabolites and are easily eliminated from the body. Enzymes such as glutathione-s-transferase (GST), UDP-glucuronosyl transferase (UGT), NAP (P) H: quinone oxidoreductase (NQO1), thioredoxin reductase 1 (TR1), and heme oxygenase 1 (HO-1) mediate during phase 2 metabolism conjugation and then the detoxification of potential carcinogens [50]. It is argued that ITCs taken from cruciferous vegetables play a role in inhibiting phase 1 enzymes and inducing phase 2 enzymes; thus, they have an anticarcinogenic effect by reducing the activation of procarcinogens and eliminating carcinogens [50, 84]. In a study conducted on rats, 40 *μ*mol/kg/day of SFN treatment was found to increase GST and NQO1 activities in the duodenum, stomach, and bladder tissues [85]. In cell culture and animal studies, it is stated that stable ITCs (such as SFN, AITC, PEITC, and BITC) have an anticarcinogenic effect by inhibiting CYP enzymes, while indoles (I3C, DIM) activate phase 2 enzymes as well as inhibit CYP enzymes [65]. Nakajima et al. explain that PEITC has a chemopreventive effect against nitrosamine-induced carcinogenesis by causing inhibition of CYP450 enzymes [86].

Genes encoding phase II enzymes contain antioxidant response element (ARE) regions. It has been reported that ITCs phosphorylate ERK1/2 and JNK1/2 and accordingly Nrf2 in cells, resulting in translocation activation in the Nrf2/ARE signalling pathway and an increase in phase II enzyme expression [61, 85]. In a randomized controlled double-blind study, it was observed that the excretion of benzene and acrolein (tobacco smoke substances) mercapturic acids increased in urine after the oral administration of PEITC to smokers for 5 days [87].

Clinical studies have shown that dietary isothiocyanates induce the activities of plasma GST-A and peripheral lymphocyte GSTM, and this effect is more significant in females than in male subjects. It was determined that colonic mucosal GST activity increased with the increase of lymphocyte GST activity [88]. Glutathione transferase (GST), one of the phase 2 enzymes, plays an important role in the detoxification of environmental mutagens, but it also metabolizes anticarcinogenic phytochemicals [27]. GSTs, which are the primary enzymes involved in isothiocyanate metabolism, conjugate isothiocyanates with glutathione, resulting in the formation of less reactive metabolites that are more easily excreted in the urine [89]. It has been determined that the elimination of ITCs is slower, and the exposure time is longer in individuals with no or low GST activity, and they also maintain their biological activities for a longer time [65]. Decreased expression of GST is expected to result in a longer biological half-life of isothiocyanates and increased cancer chemopreventive activity [88]. It is indicated that individuals with homozygously silenced GSTM1 and GSTT1 genes will metabolize isothiocyanates at a slower rate; therefore, they may be more exposed to isothiocyanates after consuming cruciferous vegetables [19, 89]. A case-control study examining the relationship between exposure to isothiocyanate, and colorectal cancer found an inverse relationship between urinary isothiocyanates and colorectal cancer risk [19]. The protective effect of isothiocyanates was most evident among individuals whose GSTM1 and GSTT1 genes were silenced. However, no significant relationship was found between isothiocyanate concentration and risk of colorectal cancer among individuals carrying the GSTM1 or GSTT1 gene [19].

### 3.6. Cell Cycle Inhibition and Apoptosis

After a cell divides, it goes through a series of stages known as the cell cycle before dividing again [61]. Apoptosis or programmed cell death is a highly regulated process that occurs under a variety of physiological and pathological conditions as part of the cellular mechanism. In addition to the development and maintenance of homeostasis, apoptosis plays an important role in the destruction of cells that are damaged or no longer needed in the organism. However, improper regulation of apoptosis can cause serious disorders, especially cancer [50]. ITCs, an important glucosinolate of cruciferous vegetables, are reported to regulate apoptosis mainly through the mitochondrial release of cytochrome c, Bcl-2 family regulation, MAPK signalling, and subsequent activation of caspases, a family of cysteine proteases responsible for initiation and execution of apoptosis [50]. It is known that sulforaphane, an essential component of these vegetables, may be a dietary factor preventing cancer with many mechanisms. Among these mechanisms, sulforaphane inhibits cell proliferation and increases apoptosis [90]. It has been emphasized that sulforaphane inhibits tumour growth by inducing caspase and mitochondrial pathway apoptosis and stopping the cell cycle in the G2/M phase and may be an effective agent in the prevention of colorectal cancer [91]. In addition to sulforaphane, isothiocyanates such as I3C, phenethyl isothiocyanate, benzyl isothiocyanate, and allyl isothiocyanate have been shown in cell culture models to have similar effects on cell apoptosis. However, considering the metabolism and bioavailability of these components, there is no clear data on physiological effects in humans following oral ingestion [61, 92]. Therefore, human-based studies are needed to better understand the effects of glucosinolates in preventing colorectal cancer.

Tumourigenesis is a complex process that lasts from tumour initiation to metastasis and includes many stages. Tumour cells are characterized by the result of uncontrolled proliferation processes in which cell division occurs more rapidly. In addition to proliferation, other molecular mechanisms affected are programmed cell death/apoptosis and the cell cycle [93]. Besides activation of detoxification enzymes and apoptosis, another mechanism of glucosinolates associated with colorectal cancer is associated with the suppression of tumourigenesis [94]. Colorectal cancer develops in a complex and multistage process involving gradual disruption of homeostatic mechanisms that control epithelial proliferation, inflammation, and differentiation. One of these disruptions is the activation of the Wnt/*β* catenin signalling pathway, which has an important role in the tumourigenesis of colorectal cancer in humans [95]. The signalling process by the Wnt family of glycolipoproteins secreted through the transcription coactivator *β*-catenin controls embryonic development and adult homeostasis. Mutations in the Wnt pathway are generally associated with some diseases, especially cancer [96]. Loss-of-function mutations in adenomatous polyposis coli found in nearly all human colorectal cancers or mutations in *β*-catenin at the phosphorylation site lead to stabilization of the *β*-catenin protein and abnormal activation of Wnt/*β*-catenin signalling [97]. Accumulation of overexpressed *β*-catenin in the nucleus activates the expression of its target genes, such as cyclin D1, which is required for the G1/S transition in the cell cycle. This transition contributes to cell proliferation and tumourigenesis in colorectal cancers [98–101].

Isothiocyanates found in cruciferous vegetables are known to inhibit the proliferation of colorectal cancer cells [94]. It has also been reported that isothiocyanates reduce *β*-catenin binding of the cyclin D1 promoter to a positive cis-element, thereby inhibiting *β*-catenin-dependent cyclin D1 transcription, possibly through a direct interaction between p65 and *β*-catenin. Although targeting the *β*-catenin/cyclin D1 pathway in the prevention of colorectal cancer is considered a promising strategy, more research is needed to elucidate further mechanisms [97].

It is stated that ITCs also block DNA replication in noncancerous cells. However, DNA damage is more powerful in cancer cells than in noncancerous cells due to inadequate DNA repair. As a result, the selective antiproliferative impact of ITCs against cancer cells is related to cancer cells' less efficient DNA repair compared to normal cells [102].

### 3.7. Antiangiogenesis and Antimetastasis

Angiogenesis contributes to the growth, invasion, and metastasis of cells by contributing to the increased nutritional and oxygen requirements of cancer cells. Metastasis is a complex process of increased invasiveness, circulation, colonization of tissues, and proliferation. The metastatic process is mainly regulated by genes that play an important role in the interaction of angiogenesis with the extracellular matrix [103]. The angiogenesis process is controlled by angiogenic factors such as MMPs, plasminogen activators (PAs), integrins, and vascular endothelial growth factor (VEGF). The imbalance between angiogenic factors and angiogenesis inhibitors plays an important role in carcinogenesis [103].

Hypoxic conditions induce tumour growth and metastasis by stimulating angiogenesis. Stimulation of angiogenesis is regulated by the activation of the mTOR signalling pathway and the increase in hypoxia-inducible factor-1*α* (HIF-1*α*) and VEGF expression. In an in vitro study, the efficacy of sulforaphane treatment in HCT116 colon cancer cells under hypoxic conditions was examined, and it was observed that sulforaphane treatment dose-dependently suppressed HIF-1-regulated gene expressions such as VEGF, HO-1, and GLUT1. These data findings suggest that sulforaphane can inhibit human colon cancer angiogenesis and progression by inhibiting HIF-1*α* and VEGF expression under hypoxic conditions [104].

Overexpression of the COX enzyme and resultant overproduction of prostaglandin E2 (PGE2) are also linked to colorectal cancer. Enhanced PGE2 signaling promotes VEGF expression and, as a result of this, cancer cell proliferation, metastasis, and angiogenesis can occur [105]. Matrix metalloproteinases (MMPs) are overexpressed in almost all human malignancies, in addition to PGE2 overproduction. Matrix metalloproteinase-2 (MMP-2), MMP-9, and urokinase-PA (u-PA) are enzymes that degrade extracellular matrix components and play a key role in cancer invasion and metastasis. As a result, anticancer treatment focuses on inhibiting MMP, u-PA, and COX-2 activity. BITC suppressed MMP-2 and MMP-9 mRNA expression and protein levels in HT29 cells, according to Lai et al. COX-2, iNOS, and u-PA, a serine protease that plays a role in tumour invasion, proliferation, and metastasis, were also suppressed by BITC. The phosphorylation of Akt and JNK is also reduced by BITC. Overall, it was determined that BITC inhibits migration and invasion by downregulating PKC and then blocking the MAPK and PI3K/Akt signaling pathways, as well as NF-B and uPA, culminating in MMP-2 and MMP-9 inhibition [106]. According to another study, AITC also prevented the metastasis of EGF (epidermal growth factor)-stimulated HT29 cells through reducing MAPK signaling pathways. EGF-stimulated activation of the EGFR pathway enhances colorectal cancer cell proliferation, invasion, metastasis, and cell death suppression. EGFR is autophosphorylated in response to EGF stimulation, which activates intracellular signaling pathways such as MAPKs (ERK, p38, and JNK) [79].

In the presence of PEITC at pharmacologically relevant doses (1 mol/L), human umbilical vein endothelial cells (HUVEC) in vitro neovascularization and migration (invasion potential) were markedly reduced. PEITC-mediated reduction of angiogenic characteristics of HUVEC in vitro was linked to decreased VEGF production, decreased VEGF receptor 2 protein levels, and inactivation of the prosurvival serine-threonine kinase Akt. HUVEC survival was reduced by PEITC treatment in a concentration- and time-dependent manner. Consequently, interfering with EGFR, inhibition of VEGF, MMPs, COX-2 or downstream intracellular signalling by ITCs might therefore be used as a cancer chemotherapeutic approach [107]. However, because the current studies on this subject are in vitro studies, it is impossible to generalize the effects to humans and determine the optimal dose and duration of usage.

### 3.8. Toxicity and Possible Side Effects of Glucosinolates

Although many beneficial effects of glucosinolates such as cancer prevention, inflammation, stress response, phase I metabolism, and regulatory functions in antioxidant activities have been shown, some side effects have also been observed in animals fed diets containing high glucosinolate like reduced feed intake and growth, gastrointestinal irritation, goiter, anemia, hepatic, and renal lesions [108]. A high intake of sulfur in the diet can cause deficiency by limiting the absorption of copper and other trace minerals [109]. In addition, high sulfur intakes in ruminants have been associated with polio [110].

The majority of studies of the toxicity level of glucosinolates refer to animal models with dietary doses exceeding normal human daily consumption, yet there is little epidemiological evidence and toxicological data on toxicity in humans [111]. There are several studies highlighting toxicity cases such as the goitrogenic and mutagenic effects of ITCs [112, 113]. Additionally, some adverse activities of hydrolysis by-products such as nitriles, thiocyanates, goitrin, epithionitriles, and cyanides have been demonstrated [62, 114].

## 4. Phenolic Compounds

Sulfur vegetables have many phytochemical compounds beneficial for health in addition to the nutrients they contain [33]. Phytochemicals are secondary metabolites found naturally in plants and act on cells with various molecular targets [115]. Phenolic compounds, which are of great importance among phytochemicals, are divided into many subgroups, including flavonoids, flavones, flavone 3-ols, anthocyanidins, isoflavonoids, chalcones, phenolic acids, and lignin [116]. Tannins, phenolic acids, anthocyanidins, flavonols, coumarins, and flavones are phenolics commonly found in cruciferous vegetables [32]. In the study, the bioactive components of 12 different types of sulphurous vegetables were examined. Of the flavonoids, ferulic acid, cinnamic acid, caffeic acid, and p-coumaric acid were observed in almost all vegetables. The vegetables containing the most bioactive compounds are Brussels sprouts and cabbage. Arugula, watercress, and Brussels sprouts showed the highest antioxidant activity [46].

### 4.1. Chemical Structure

Examination of phytochemicals in sulphureous vegetables revealed that the most common phenolic compounds are simple phenols, phenolic acids, flavonoids, and hydroxycinnamic acids. Flavonoids have a diphenyl propane (C6-C3-C6) skeleton and vary according to the number and arrangement of hydroxyl groups in their structures and the alkylation and glycolysis of these groups [117]. Among the flavonoids that vegetables contain are pelargonidin, cyanidin, delphinidin, peonidin, petunidin, and malvidin [33]. Hydroxycinnamic acids are found in tissues in various conjugated forms with esters and amides, and rarely as free acids [117]. The majority of hydroxycinnamic acids in the structures of sulphurous vegetables are p-coumaric, synaptic, and ferulic acids and are usually found together with sugar or other hydroxycinnamic acids [33, 118].

### 4.2. Metabolism and Bioavailability

The absorption of phenolic compounds varies according to the sugar and organic acids in their structure, the structural properties of the compound, its molecular weight, whether it is hydrophilic or hydrophobic, and the presence of the group capable of hydrogen bonding [119]. They are decomposed and metabolized by conjugation reactions in the stomach, small intestine, or colon microflora. Phenolics are first hydrolysed to their free aglycones, then conjugated by methylation, sulfation, glucuronidation, or a combination thereof [120]. While low molecular weight gallic acid and isoflavones are easily absorbed, many phenolic compounds can pass into plasma at low rates [119].

### 4.3. Health Promotion and Cancer Prevention

Phenolic compounds show antioxidant activity in the body due to their free radical capture capacity and are associated with many diseases [121]. They also prevent oxidative DNA damage by inhibiting the binding of carcinogens to DNA. Therefore, it has protective effects against cancer [116].

Flavonoids are powerful antioxidants with antibacterial, antiviral, and anti-inflammatory properties that are effective in protecting cells against cancer development. Apigenin, one of the flavonoids and found in some sulphurous vegetables, exhibits chemopreventive and cytostatic properties, reduces angiogenesis, inhibits colorectal cell growth, and induces apoptosis [122]. In the study, it was determined that proapoptotic proteins (NAG-1 and p53) and cell cycle inhibitors (p21) were induced in the presence of apigenin in vivo and in vitro. In addition, the increased p53 activation through phosphorylation in animal models reduces the number of polyps associated with it [123]. Similarly, it increased the cell surface CD26 level in human colorectal cancer cells and inhibited tumour metastasis pathways [124]. It has been noted that kaempferol in broccoli also shows antitumoural activity in colorectal tumour lines, inhibits the activity of CDK2, CDK4, and Cdc2, and the cell cycle by inducing apoptosis [122]. Quercetin, which is one of the flavanols and is found in many types of vegetables, has an antiproliferative effect. In the study, it was found that it inhibits the expression of the Ras p21 protein activator at the cell level and prevents Ras activation [125]. A diet rich in quercetin inhibits cancer formation by acting on apoptosis, angiogenesis, cell proliferation, autophagy, and inflammation through various molecular pathways [126].

It is seen that different phenolic compounds contained in sulphurous vegetables are effective in preventing cancer through varying pathways. Sulfurous vegetables have an important place in the prevention of cancer since bioactive molecules and nutrients are found in complex forms in foods and the intake of these substances into the body in organic form is more effective.

### 4.4. Therapeutic Roles

Although phenolic compounds influence the prevention of cancer formation, their bioavailability and interaction with other therapeutic agents pose a problem when used in therapy [127]. Quercetin and genistein, which are compounds contained in sulfurous vegetables, have been applied in animal and cell models, but used only in few clinical studies. In the study, genistein was tested in colorectal cancer cells, and it was seen that it inhibited cell proliferation and induced apoptosis. Genistein inhibited Akt phosphorylation in HCT-116 cells while increasing the mRNA and protein levels of Bax [128]. In a similar study, it was concluded that genistein showed anticancer properties on HT29 colon cancer cells by modulating the caspase-3 and p38 MAPK pathway at different transcriptional and protein levels [129]. Quercetin and other phenolic compounds have effects on programmed cell death and cellular senescence. The study has revealed that quercetin induces apoptosis in colon cancer cells, increases Bax immunoreactivity, and decreases Bcl-2 activity [130].

With nanotechnological developments, formulations that provide controlled release of bioactive compounds and increase bioavailability can be developed [127]. Thus, phenolic compounds whose effects have been determined can be used as an alternative treatment in cancer treatment and suppression of tumour growth. In addition, consuming vegetables rich in phenolic compounds can create a natural therapeutic effect.

## 5. Tocopherols

### 5.1. Chemical Structure

Tocopherols and tocotrienols, called vitamin E, have 8 different structures and are synthesized by plants and other photosynthetic organisms. Different tocopherols are distinguished from each other by the number and position of methyl groups attached to the nucleus. Essential for humans, *α*-tocopherol is the most abundant in nature and has the highest biological activity. Tocotrienols are distinguished from tocopherols by the presence of three double bonds in the side chains. Only *α* and *β*-tocotrienol appear to have significant vitamin activity [131, 132].

### 5.2. Metabolism and Bioavailability

Dietary tocopherols are absorbed from the intestine and enter the circulation with chylomicrons. Ester forms are hydrolysed in the intestine and absorbed in free form. *α*-tocopherol reachs the liver where it is included in the structure of low-density lipoproteins, and other forms are released with bile acids [133]. Nutritional factors and the physiological and health status of the individual affect the uptake of tocopherols [134]. While there is no effect of dietary fiber, bioavailability increases as chylomicron formation will increase when taken together with fats [131]. The content of *α*-tocopherol in sulfur vegetables such as broccoli, Brussels sprouts, cabbage, cauliflower, and Chinese cabbage differs among cultivars [135].

### 5.3. Health Promotion and Cancer Prevention

Tocopherols display many different biological activities such as antioxidant capacity, modulation of immune function, regulation of cell differentiation, and proliferation [134]. It was thought that low vitamin E intake and nutritional status increased the risk of cancer, and *α*-tocopherol supplementation in deficient groups was thought to reduce cancer risk. However, in recent years, it has been observed that *γ* and *δ*-tocopherols show stronger anticancer activity compared to *α*-tocopherol [136, 137]. In the study, a diet containing *γ*-tocopherol decreased the level of RAS p-21 in rats. Therefore, it has been suggested that *γ*-tocopherol may play a role in the prevention of colon cancer [138]. When studies with different forms of vitamin E were examined, it was noted that *γ*-tocopherol, *δ*-tocopherol, *γ*-tocotrienol, and *δ*-tocotrienol had a high anticancer effect. Compared to *α*-tocopherol, these forms have been stated to be more effective in inhibiting cancer formation pathways such as cyclo-oxygenase (COX)- and 5-lipoxygenase (5-LOX)-catalyzed eicosanoids, nuclear transcription factor-*κ*B (NF-*κ*B), and signal transducer and transcription factor 3 (STAT3) [139]. In studies on animals and humans, *γ*-tocopherol in cells has been found to protect cells against inflammatory damage, but epidemiological studies have shown that its effect is complex [140]. *δ*- Tocopherol prevents colon carcinogenesis by reducing the proinflammatory response induced by reactive oxygen species. Although *β*-tocopherol shows antioxidant activity like *α*-tocopherol, it does not inhibit protein kinase C activity, cell proliferation, and gene expression. It does not affect IL-1*β* release and caspase-3 activity. Moreover, it is 10 times less active than *α*-tocopherol in inhibiting thrombin-induced PKC activity [141]. In addition, in meta-analysis studies examining the effectiveness of vitamin E in colorectal cancer, it was concluded that the use of high-dose supplements is not protective against cancer [142]. Although the biological activity of *α*-tocopherol, one of the tocopherols, is very high and has a direct effect on vitamin E deficiency, it is observed that *γ*-tocopherol has great importance in the prevention of cancer.

### 5.4. Therapeutic Roles

Thanks to the anti-inflammatory and anticarcinogenic effects of tocopherols, research is being conducted on their use in cancer treatment. It is reported that different forms of vitamin E activity play a role in the treatment and control of cancer by increasing the sensitivity of cancer cells to chemotherapeutic drugs and the effectiveness of treatment [139, 143]. In the study, it was determined that *γ*-tocopherol-rich tocopherol mixture inhibited colon carcinogenesis in mice treated with azoxymethane (AOM)/dextran sulfate sodium (DSS). It has been shown that this effect may be due to the apoptosis-inducing, anti-inflammatory, antioxidative, and reactive nitrogen species capturing activities of tocopherols [144]. It has been noted that combination therapy with 5-fluorouracil and *γ*-tocopherol in HT-29 colon cancer cells stimulates apoptosis and can be used as a new therapeutic approach that can increase the effectiveness of chemotherapy [145]. It is seen that *γ*-tocopherol is significant in the treatment of colorectal cancers. Existing studies are generally at the cell and animal level, and clinical and epidemiological study results are needed to determine therapeutic doses.

## 6. Carotenoids

### 6.1. Chemical Structure

Carotenoids are the isoprenoid group of pigmented compounds and are synthesized by plants and microorganisms [146]. They are chemically polyisoprenoid and have a long chain of conjugated double bonds and a symmetrical appearance around the central double bond [147]. According to their functional groups, they are divided into xanthophylls and carotenes. Xanthophylls contain oxygen as a functional group, and lutein and zeaxanthin are in this group. Carotenes such as *α*-carotene, *β*-carotene, and lycopene consist only of the main hydrocarbon chain. Depending on the changes in their chemical structure, the polarity of carotenoids changes, and their biological functions are affected [148].

### 6.2. Metabolism and Bioavailability

Although there are many different types of carotenoids in nature, only 20 of them have been observed in our blood and tissues. About 90% of the carotenoids in the human body and diet are composed of *α*-carotene, *β*-carotene, lutein, and cryptoxanthin [147]. The bioavailability of carotenoids is affected by many factors such as their release from the nutrient matrix, their solubility in the digestive system, and their absorption from intestinal epithelial cells. Due to their hydrophobic nature, their absorption from the gut is limited. Oils increase the dissolution and bioavailability of carotenoids as mixed micelles. Intake of carotenoids dissolved in micelles by intestinal cells is provided by simple diffusion or facilitated diffusion [149]. Cabbage and leafy greens are sources of dietary carotenoids. The amount of carotenoid they contain may vary depending on the region where they are grown, exposure to the sun, and the type of agriculture applied [135].

### 6.3. Health Promotion and Cancer Prevention

Carotenoids are the main group of plant pigments that play an important role in the prevention and cure of different diseases, including cancer. Thanks to their antioxidant effects, carotenoids show anticancer properties in various ways such as anti-inflammation, antiangiogenesis, immunomodulation, triggering cell differentiation, and antiproliferation [150]. Therefore, vegetables with high carotenoid content have anticancer effects [151]. Increasing the amount of serum *β*-carotene reduces disease mortality [152]. There is also an inverse relationship between *α*-carotene intake and cancer formation [151]. Major carotenoids such as *β*-carotene, *β*-cryptoxanthin, and lycopene show chemopreventive properties in preventing cancer by suppressing ROS, inducing peroxyl radicals, and phase II enzymes [150].

An epidemiological study found an inverse relationship between the risk of colorectal adenoma and consumption of *β*-carotene, lycopene, lutein, and zeaxanthin in 29,363 men undergoing intestinal endoscopy [153]. A significant inverse relationship was found between serum concentrations of carotenoids and the presence of colorectal polyps in 893 individuals who underwent colorectal endoscopy. Carotenoids found in green-yellow vegetables have been reported to have protective effects against colorectal neoplasm [154]. The proliferation of HT-29 colon cancer cells was reduced when 2 *μ*M or more of lycopene was used in the cell culture study. Similarly, lycopene suppressed COX-2, PGE2, and phosphorylated ERK1/2 protein in tumour-developed mice while increasing PCNA and p21 expression [155, 156]. The results show that carotenoids reduce tumour growth and colorectal cancer progression. However, it has been reported that carotenoids do not have a protective effect against cancer when used as a supplement [151].

Lutein (*β*, *ε*-carotene-3, 3′-diol.) and its stereoisomer zeaxanthin are both xanthophyll carotenoids. Lutein is present in a variety of green leafy cruciferous vegetables, such as kale (21,900 *μ*g/100 g) and mustard greens (9,900 *μ*g/100 g), where the yellow-orange colour of this xanthophyll carotenoid is masked by the dominant green colour of chlorophyll [157]. Interestingly, from a pool analysis of 11 cohort studies findings showed that the dietary intake of lutein could not be associated with colorectal cancer risk [158]; however, noted that this subject required further investigation to be found conclusive.

### 6.4. Therapeutic Roles

Found in sulfurous vegetables, *β*-carotene is one of the important carotenoids in the diet, which has antioxidant properties. In experimental studies, *β*-carotene induces cell cycle arrest and apoptosis in human colon adenocarcinoma. It also inhibits the production of LPS-stimulated inflammatory mediators by suppressing the activation of the NF-*κ*B pathway [159]. In the study, *β*-carotene supplementation inhibited tumourigenesis and expression of M2 macrophage markers in a mouse model of dextran sodium sulfate-induced colitis-associated colorectal cancer. It has been reported that *β*-carotene has a potent therapeutic effect in colorectal cancer [160]. *β*-carotene has also been found to regulate NF-*κ*B activation through a redox mechanism to induce growth inhibition and apoptosis in human colon adenocarcinoma cells [161]. However, when meta-analysis studies were reviewed, it was seen that there was not enough evidence for the use of *β*-carotene as a supplement alone or in combination with other compounds in the treatment of colorectal cancer [142]. The pharmacological use of carotenoid compounds is limited due to their low bioavailability and solubility, as they are often lipophilic compounds. Encapsulation of carotenoids in different nanocarriers increases their solubility, cellular uptake, membrane permeability, bioaccessibility, and stability, making them usable in cancer therapy [150].

## 7. Other Essential but Important Compounds

### 7.1. Fibers

Like most other vegetables, cruciferous vegetables are good sources of various nutrients and phytochemicals such as glucosinolates, flavonoids, fiber, and lignans that contribute to health promotion [162]. Sulfurous vegetables contribute significantly to dietary fiber intake [25]. Among sulfurous vegetables, kale is known for its relatively highest fiber content with 4.6% (w/w on a fresh weight basis), then comes broccoli (w/w 30.4% dry weight) and cauliflower (26% w/w dry weight basis, 26, 7%) [25, 163, 164] (Table 8).

The American Dietary Guidelines 2015–2020 stated that adults should consume at least 2.5 cups/vegetable per day from all five vegetable groups (dark green, red, and orange, starchy vegetables, legumes, and others) for a 2000 calorie diet. The guideline does not set a separate recommendation for cruciferous vegetables but recommends that adults consume the equivalent of 1½–2½ cups per week of dark green vegetables, including cruciferous vegetables, as part of a healthy meal [22]. The guidelines inform that 1 cup of raw or cooked vegetables such as broccoli, cauliflower, and Brussels sprouts are equivalent to 1 cup of a vegetable portion. 2 cups of raw leafy greens such as kale and Chinese cabbage are equivalent to 1 cup of a vegetable portion [22]. Meta-analysis reports of observational studies show an inverse relationship between fiber intake and colorectal cancer risk [165–167]. A recent review of previous meta-analysis studies obtained similar conclusions [168]. In this context, it can be said that a portion of sulfurous vegetables such as Brussels sprouts, broccoli, cabbage, and cauliflower contribute significantly to daily fiber intake.

### 7.2. Seed Oils

The negligible amount of fat (less than 1.0%) in cruciferous vegetables makes them a significant component of a low-fat and heart friendly diet. Watercress has the relatively highest amount of fat (∼1%), while most other cruciferous vegetables lack dietary fat [164]. In vegetables and fruits, in addition to flavonoids [169] and polyphenols [170, 171], phytosterols also have a protective effect against cancer [172]. Phytosterols are derivatives of the 4-desmethyl sterol molecule and are only found in plant foods. They are alcohols of 28 or 29 carbons, structurally similar to 27-carbon cholesterol [173]. The most common phytosterols in the human diet are *β* sitosterol, campesterol, and stigmasterol. All phytosterols contain a double bond at the 5th carbon atom, and when this double bond becomes saturated under certain conditions, these compounds are called plant stanols; two of the most common ones are *β*-sitostanol and campestanol [174]. Oils, nuts, legumes, grains, fruits, and vegetables are the main dietary sources of phytosterols [175, 176]. Regarding their bioactivity, phytosterols have also been suggested and experimentally confirmed to reduce cancer risk as well as lower serum cholesterol levels [177], mediating cell cycle arrest, inducing cellular apoptosis [178], and reducing cellular oxidative stress [179]. Epidemiological studies have shown that dietary intake of total phytosterol, *β*-sitosterol, campesterol, and campestanol is inversely related to colorectal cancer risk [180]; beta-sitosterol and stigmasterol are inversely related to colon cancer risk [181]. On the other hand, a case-cohort study conducted in the Netherlands revealed no reduction in colorectal cancer risk due to high intakes of any of the phytosterols [182]. Sulfurous vegetables are a good source of phytosterols and terpenoids known for their anticancer and cardioprotective activities. Brassicasterol is another sterol found in broccoli and other sulfurous vegetables. The content of sterols differs in parts of plants propagated and cultivars. The flowers contain relatively higher amounts of sterols compared to the stems, and a maximum of 230 mg/100 g dw (100 gram at dry weight) sterols has been reported [47]. It is known that storing vegetables in a controlled atmosphere (5% CO_2_ + 2% O_2_ and 93% air) prevents the degradation of sterols compared to normal conditions (0% CO_2_ + 21% O_2_ and 79% air). Therefore, it is recommended to use a controlled atmosphere to obtain maximum health benefits from sterols and other phytochemicals in sulfurous vegetables [49]. Other bioactive compounds found in sulfurous vegetables such as phloroglucinol, oleuropein, oleacein, sargachromanol, epifriadelanol, terpenoids, and omega-3 fatty acids also have antioxidant/antisenescent properties, reduce the amount of ROS and lipid peroxidation products, and inhibit the expression of matrix metalloproteinases [183].

### 7.3. Vitamin K

Vitamin K is a group of fat-soluble essential nutrients that exists in two natural forms (vitamin K_1_ or phylloquinone, present in green leafy vegetables including cruciferous ones, and vitamin K_2_ or menaquinone, synthesized by intestinal flora). Menadione is a catabolic product of vitamin K (phylloquinone and menaquinone) and urinary menadione (conjugates, glucuronides, and sulphates of the reduced form of menadione, menadiol) mirrors the dietary phylloquinone intake [184]. Menadione reduces the numbers and incidences of 1,2-dimethylhydrazine-induced colon tumours in mice, but the mechanism of anticancer activity of menadione in colorectal cancer is not very clear [185].

## 8. Conclusion

Cruciferous vegetables are a significant source of bioactive metabolites as well as their nutritional properties. With these features, they show a significant interaction with colorectal cancer, which is one of the top public health and clinical issues today. In the present review, bioactive components of cruciferous vegetables, which were the most discussed in the literature regarding colorectal cancer, were examined. In this context, it can be concluded that many metabolic and genomic pathways and potential mechanisms regarding these vegetables and their bioactives can both prevent colorectal cancer, as well as reduce its incidence rates, and so would have a therapeutic effect.

Although a large number of studies have been conducted on the effects of these vegetables and their bioactive metabolites in preventing cancer in the literature, it can be seen that more data are needed regarding their therapeutic effects (in terms of appropriate consumption dose, extraction technique, possible side effects, etc.). Because when the accumulated literature is analyzed, it seems the majority of these studies are geared towards more preventive roles (both in primary prevention and secondary prevention/slowing progression) instead of their therapeutic roles. Moreover, there are many in vitro studies specifically focused on their therapeutic effects; however, these results need to be supported by more human clinical studies. This should allow for a lot of issues in relation to their bioactive components' synergistic or antagonistic effects to be clarified.

These vegetables can be used both for natural nutrition and have the potential to be a natural medicine that can be used by extracting bioactive metabolites. Both from a dietetic and pharmacological point of view, these features make them very important for adequate/balanced and clinical nutrition and point out that they should be included more in our diet.

## Figures and Tables

**Table 1:** Common name and botanical classification of cruciferous vegetables.

Common name	Botanical classification
Broccoli	*B. oleracea convar. botrytis var. cimosa* (*commonly var. italica*)
Brussels sprouts	*B. oleracea var. gemmifera*
Cabbage, white	*B. oleracea convar. capitata var. alba*
Cabbage, red	*B. oleracea convar. capitata var. rubra*
Cabbage, savoy	*B. oleracea convar. capitata var. sabauda*
Cauliflower	*B. oleracea convar. botrytis var. botrytis*
Chinese cabbage	*B. rapa ssp. pekinensis*
Kale	*B. oleracea convar. acephala*
Kohlrabi	*B. oleracea var. gongylodes*
Mustard greens	*B. juncea*
Pak choi	*B. rapa ssp. chinensis*
Portuguese tronchuda cabbage	*B. oleracea var. costata*
Radish	*Raphanus sativus*
Rutabaga	*B. napus var. napobrassica*
Turnip	*B. rapa var. rapa*
Arugula (rocket)	*Eruca sativa*
Collards	*Brassica oleracea var. sabellica*
Horseradish	*Armoracia rusticana*

**Table 2:** Nutritive composition of cruciferous vegetables (100 g, raw).

Cruciferous vegetables	Water (g)	Energy (kcal)	Protein (g)	Fat (g)	Carbohydrate (g)	Fiber (g)	Ca (mg)	Fe (mg)	Mg (mg)	P (mg)	K (mg)	Na (mg)	Zn (mg)	Cu (mg)	Mn (mg)	Se (*μ*g)
Broccoli	90	39	2.57	0.34	6.27	2.4	46	0.69	21	67	303	36	0.42	0.059	0.197	1.6
Cabbage	92.2	25	1.28	0.1	5.8	2.5	40	0.47	12	20	170	18	0.18	0.019	0.16	0.3
Cauliflower	92.1	25	1.92	0.28	4.97	2	22	0.42	15	44	299	30	0.27	0.039	0.155	0.6
Brussels sprouts	86	43	3.38	0.3	8.95	3.8	42	1.4	23	69	389	25	0.42	0.07	0.337	1.6
Kale	89.6	35	2.92	1.49	4.42	4.1	254	1.6	33	55	348	53	0.39	0.053	0.92	0.9
Arugula	91.7	25	2.58	0.66	3.65	1.6	160	1.46	47	52	369	27	0.47	0.076	0.321	0.3
Chinese cabbage	95.3	13	1.5	0.2	2.18	1	105	0.8	19	37	252	65	0.19	0.021	0.159	0.5
Collards	89.62	32	3.02	0.61	5.42	4	232	0.47	27	25	213	17	0.21	0.046	ND	1.3
Horseradish	85.08	48	1.18	0.69	11.29	3.3	56	0.42	27	31	246	420	0.83	0.058	ND	2.8
Radishes	95.3	16	0.68	0.1	3.4	1.6	25	0.34	10	20	233	39	0.28	0.05	0.069	0.6
Rutabagas	89.4	37	1.08	0.16	8.62	2.3	43	0.44	20	53	305	12	0.24	0.032	0.131	0.7
Turnips	91.9	28	0.9	0.1	6.43	1.8	30	0.3	11	27	191	67	0.27	0.085	0.134	0.7
Watercress	95.1	11	2.3	0.1	1.29	0.5	120	0.2	21	60	330	41	0.11	0.077	0.244	0.9
Kohlrabi	91	27	1.7	0.1	6.2	3.6	24	0.4	19	46	350	20	0.03	0.129	ND	0.7

ND, No data.

**Table 3:** Vitamins and bioactive components of cruciferous vegetables (100 g, raw).

Cruciferous vegetables	Vitamin C (mg)	Folate (*μ*g)	Vitamin A (*μ*g)	Vitamin E (mg)	Vitamin K (*μ*g)	Beta-carotene (*μ*g)	Lutein + zeaxanthin (*μ*g)	Glucosinolate (mg)	Sulforaphane (*μ*g)^*∗*^
Broccoli	91.3	65	8	0.15	102	93	745	1297	260
Cabbage	36.6	43	5	0.15	76	42	30	1069	10.1
Cauliflower	48.2	57	0	0.08	15.5	0	1	1178	ND
Brussels sprouts	85	61	38	0.88	177	450	1590	1013	2.6
Kale	93.4	62	241	0.66	390	2870	6260	1206	1736–3027^a^
Arugula	15	97	119	0	109	1420	3560		110
Chinese cabbage	45	66	223	0.09	45.5	2680	40	297	540
Collards	35.3	129	251	2.26	437.1	2991	2323	11.4–36.4^b^	ND
Horseradish	24.9	57	0	0.01	1.3	1	10	8.9–12.5^b^	ND
Radishes	14.8	25	0	0	1.3	4	10	676	16
Rutabagas	25	21	0	0.3	0.3	1	19	458	ND
Turnips	21	15	0	0.03	0.1	0	0	698	60
Watercress	43	9	160	1	250	1910	5770	6–28^b^	ND
Kohlrabi	62	16	2	0.48	0.1	22	0	829	ND

^*∗*^ *μ*g/g dry weight, ^a^frozen sample, ^b^*μ*mol/g dry weight, ^c^*μ*mol/100g wet weight, and ^d^mmol ND: no data.

**Table 4:** Epidemiological studies on cruciferous vegetables and colorectal cancer.

The characteristic of participants	Dietary specifications	Follow-up	Main outcomes	References
Netherlands cohort study on diet and cancer; 62,573 women and 58,279 men aged 55–69 years	Cruciferous vegetables	6.3 years	While cruciferous vegetables have a strong negative association with colon cancer in both genders, they have been reported to increase the risk of rectal cancer in women.	[17]
Singapore Chinese Health Study; 213 cases and 1194 controls	Cruciferous vegetables	5 years	Isothiocyanates derived from cruciferous vegetables reduced the risk of colorectal cancer in individuals with low glutathione *S*-transferases.	[23]
The Western Australian Bowel Health Study; 834 colorectal cancer cases and 939 controls	*Brassica* vegetable	2 years	*Brassica* vegetables were found to be inversely related with proximal colon cancer.	
A hospital-based matched case-control study in northeast China; 833 colorectal cancer cases and 833 controls	Cruciferous vegetables	28 months	There was not a significant association between total cruciferous vegetable intake and colorectal cancer risk. In the stratification analysis by gender, higher consumption of total cruciferous vegetables in women was seen to be associated with a reduced risk of colorectal cancer.	[53]
150 colorectal cancer cases and 300 controls	Cruciferous vegetables	10 months	Cruciferous vegetables (broccoli OR = 0.11, cauliflower OR = 0.30, and cabbage OR = 0.30) were found as one of the most significant protective factors in decreasing the risk of colorectal cancer.	[52]
2390 colorectum cancer cases and 11492 controls	Cruciferous vegetables	Case–control studies in Italy and Switzerland between 1991 and 2009	Subjects who consumed at least one portion of cruciferous vegetables per week had a significantly lower risk of colorectal cancer compared to those who never or occasionally consumed them (OR = 0.83).	[51]
Chinese in Singapore; 203 colorectal cancer cases and 425 controls	Cruciferous vegetables	26 months	A protective effect of high cruciferous vegetable intake was observed in cancers of the colon and rectum combined (OR = 0.50), colon cancer, and rectal cancer (OR = 0.51).	[54]
231 colon cancer cases and 391 controls	Cruciferous vegetables	Case-control study in Utah between July 1979 and June 1983	There was an association between high cruciferous vegetable consumption and colon cancer protection in males.	[55]
163 colorectal cancer cases and 326 healthy controls	Cruciferous vegetables	Cases between 1982 and 1993	Cruciferous vegetables exhibited a significant inverse association (OR, 0.59) with cancer.	[56]
29,133 Finnish men aged 50–69 years	Cruciferous vegetables	8 years	Consumption of cruciferous vegetables was positively related to the risk of colorectal cancer.	[57]
A cohort of 17,633 white males aged 35 and older		20 years of follow-up	No clear risk patterns in terms of colon and colorectal cancer were seen for cruciferous vegetables.	[58]

OR: odds ratio.

**Table 5:** Some isothiocyanates and their glucosinolate precursors that were under investigation for their anticarcinogenic properties [61].

Glucosinolate (precursor)	ITC-indole	Food sources
Glucobrassicin	Indole-3-carbinol	Broccoli, Brussels sprouts, cabbage, cauliflower
Glucoraphanin	Sulforaphane	Broccoli, Brussels sprouts, cabbage
Gluconasturtiin	Phenethyl-isothiocyanate	Watercress
Glucotropacolin	Benzyl-isothiocyanate	Cabbage, garden cress, Indian cress
Sinigrin	Allyl-isothiocyanate	Cabbage, horseradish, mustard

**Table 6:** Some animal experimental studies on glucosinolates and colorectal cancer.

Animal characteristics	Type of intervention	Basic findings	References
ICR male mice	First group: -Azoxymethane, 15 mg/kg, once a week for 3 times -Ordinary diet or diet containing sulforaphane glucosinolates 2,200 ppm/kg/day, for 8 weeks Second group: -Azoxymethane, 15 mg/kg, once a week for 6 times -Ordinary diet or diet containing sulforaphane glucosinolates 2,200 ppm/kg/day, for 24 weeks	Daily intake of sulforaphane showed chemoprotection effects against colonic tumours in mice treated with a chemical carcinogen (azoxymethane).	[20]
ApcMin/+ mice	3 groups; control, 300 ppm sulforaphane, and 600 ppm sulforaphane for 3 weeks	ApcMin/+ mice given sulforaphane supplementation developed significantly less and smaller polyps with higher apoptotic and lower proliferative indices in the small intestine.	[21]
C57BL/6J mice	Two experimental feeding groups: AIN-93M control diet and AIN-93M with 1 *μ*mol (147.2 *μ*g) indole-3-carbinol/g diet	A diet containing indole-3-carbinol significantly reduced fecal excretion of *Citrobacter rodentium*, *Citrobacter rodentium* colonization of the colon, and reduced colon crypt hyperplasia and increased the expression of cytotoxic T cell markers CD8 and FasL mRNA.	[76]
C3H/HeN and C57BL/J6 mice infected with *Citrobacter rodentium*	Four experimental groups: uninfected mice on control diet, infected mice on control diet, uninfected mice on treatment diet (1 *μ*mol indole-3-carbinol/g diet), and infected mice on treatment diet (1 *μ*mol indole-3-carbinol/g diet)	Indole-3-carbinol significantly reduced the inflammatory response to *Citrobacter rodentium* infection by maintaining anti-inflammatory cytokine IL-22 mRNA levels while reducing expression of other proinflammatory cytokines.	[77]
C57BL/6J mice	Three experimental groups: healthy control (regular diet), disease control (regular diet), and one phenethyl isothiocyanate-diet (regular diet + 0.12% phenethyl isothiocyanate) test group	Phenethyl isothiocyanate enriched diets and reduced mucosal and submucosal inflammation and the frequency of adenocarcinoma by 17% compared to the diseased control group.	[78]

**Table 7:** Some cell culture studies on glucosinolates and colorectal cancer.

Study design	Basic findings	References
Two human colorectal cancer (p53-wild-type and p53-knockout; HCT116) cells exposed to sulforaphane in vitro and in vivo	Sulforaphane activated Nrf2-mediated antioxidant enzymes in both cells, decreased apoptotic protein expression in wild-type cells but increased in knockout cells in a dose-dependent manner, and increased the expression of a mitochondrial biogenesis marker, PGC1*α* in wild-type cells but decreased in knockout cells.	[80]
The effects of sulforaphane and a coculture with *Lactobacillus*-treated in human colon cancer HCT116 and SW480 cells and normal human colon epithelial CCD 841 CoN cells	Sulforaphane enhances apoptosis in human colon cancer cells under coculture with lactobacillus-treated peripheral blood mononuclear cells by the TNF*α* signalling pathway.	[81]
Human colorectal cancer cell lines DLD1, HCT116, HT-29, LS513, and RKO exposed to indole-3-carbinol	Multiple colorectal cancer cell types express increased CYP1A1 mRNA levels following treatment with indole-3-carbinol. Moreover, indole-3-carbinol induced a dose-dependent decrease in cell viability and apoptosis.	[82]
Human colon cancer cell lines DLD-1 and SW480 treated with different concentrations of phenethyl isothiocyanate	Phenethyl isothiocyanate significantly reduced the size and number of colorectal cancer cell spheroids and induced the apoptosis of colorectal cancer stem cells. As well, phenethyl isothiocyanate decreased the expression of cancer stem cell markers.	[83]
Human melanoma A375.S2 cell line exposed to phenethyl isothiocyanate and benzyl isothiocyanate	Sublethal concentrations of these components inhibited the mobility, migration, and invasion of A375.S2 cells. Also, they can inhibit the metastasis's expression of MAPKs, MMP-2, MMP-9, ecadherin, and NF-*κ*B in A375.S2 cells.	
Human colorectal adenocarcinoma HT29 cell line treated with allyl isothiocyanate	Allyl isothiocyanate inhibited both the invasive and migratory abilities of HT29 cells. As well as its own regulated protein levels of matrix metalloproteinase-2 and -9 and mitogen-activated protein kinases.	[79]

**Table 8:** Fiber amounts contained in cruciferous vegetables at 100 grams.

Cruciferous vegetable	Fiber g/100 g
Aragula	1.60
Bok choy	1.42
Broccoli	30.40^*∗*^
Brussels sprouts	26.94^*∗*^
Cabbage (green)	23.24^*∗*^
Cauliflower	26.70^*∗*^
Chinese cabbage	1.00
Collard greens	4.60
Daikon	<2.00
Kale	1.94
Kohlrabi	3.62
Radish	37.40^*∗*^
Turnips	1.76
Watercress	1.50

^*∗*^Dry weight basis. Values are converted from content per serving or fresh average vegetable weight.

## Data Availability

The data are available from the corresponding author on reasonable request.
